# Medicinal Extracts

**Published:** 1850-03

**Authors:** 


### MEDICINAL EXTRACTS.

We have received from Messrs. Tilden & Co., New York, specimens of various medicinal extracts prepared with great care and accuracy by themselves. These gentlemen have been actuated by an earnest desire to supply to the profession, at a moderate rate, such preparations as could be entirely relied upon. Of the narcotic extracts we can speak with confidence, having subjected them to a faithful trial. We believe them to be equal to the best imported or domestic articles. The mode of preparation adopted by Messrs. Tilden & Co., secures the peculiar active properties of the plant unaltered, at the same time that it preserves its flavor and color.

